# Insights Into the Role of Exposed Surface Charged Residues in the Alkali-Tolerance of GH11 Xylanase

**DOI:** 10.3389/fmicb.2020.00872

**Published:** 2020-05-08

**Authors:** Xiuyun Wu, Qun Zhang, Lanzeng Zhang, Shijia Liu, Guanjun Chen, Huaiqiang Zhang, Lushan Wang

**Affiliations:** ^1^State Key Laboratory of Microbial Technology, Institute of Microbial Technology, Shandong University, Qingdao, China; ^2^Taishan College, Shandong University, Jinan, China

**Keywords:** xylanase, glycoside hydrolase family 11, pH-tolerance, surface charged residues, site-directed mutation

## Abstract

Thermostable and alkaline- or acid-stable xylanases are more advantageous in agricultural and industrial fields. In this study, a rational structure-based design was conducted based on a thermostable GH11 xylanase *Tl*XynA from *Thermomyces lanuginosus* to improved pH-tolerance. Four mutant enzymes (P1, P2, P3, and P4) and five variants (N1, N2, N3, N4, and N5) were constructed by substituting surface charged residue combinations using site-directed mutagenesis. Compared to the native enzyme, two mutants P1 and P2 showed higher acid tolerance, especially at pH 3.0, presented 50 and 40% of their maximum activity, respectively. In addition, four mutants N1, N2, N3 and N4 had higher tolerance than the native enzyme to alkaline environments (pH 7.0–9.0). At pH 9.0, the residual activities of N1, N2, N3, and N4 were 86, 78, 77, and 66%, respectively. In summary, an improved pH-tolerance design principle is being reported.

## Introduction

Xylanase, as a hemicellulose-degrading enzyme, has immense application values for the utilization of hemicellulose polysaccharide resources, environmental protection, and industry (Gírio et al., 2010; Paes et al., 2012; Kumar et al., 2016; Basu et al., 2018). However, in practical terms, extreme processing conditions severely impact the enzymatic performance and restrict the application range of xylanase, include thermoalkaline environments used in textile manufacturing (Dhiman et al., 2008) and acidic environments used in feed and food industries (Knob and Carmona, 2010). Thermostable and alkaline- or acid-stable enzymes would be more advantageous in the majority of industrial processes carried out under conditions with high temperatures and extreme pH levels (Collins et al., 2005; Basu et al., 2018). Most acidstable and alkalistable enzymes are currently from extremophiles from natural systems, and these enzymes cannot meet the very large demand for industrial applications. In addition, obvious obstacles, such as the difficulty in heterologous expression of recombinant proteins need to be overcome (Zheng et al., 2018). In recent years, major efforts have been focused on increasing the operational stability of neutral xylanase owing to its greater application potential.

Xylanases are derived from a vast array of organisms, and exhibit different folding, substrate specificity, catalytic mechanisms, and physicochemical properties. Based on the CAZy database, xylanases are classified into glycoside hydrolase (GH) families 5, 7, 8, 10, 11, and 43 (Collins et al., 2005; Lombard et al., 2013). Among them, the GH11 family is considered as the only “true xylanase” family and specifically recognizes xylan substrates (Paes et al., 2012). However, most GH11 enzymes with conserved β-sandwich structures are produced by non-extremophile organisms and do not show significant operational stability in extreme conditions. Therefore, it is possible to change the pH-tolerance of these original enzymes through mutations that are based on the sequence and structural basis of acidstable and alkalistable enzymes (Bornscheuer et al., 2012; Davids et al., 2013; Madhavan et al., 2017).

The protein surface is vitally important for adapting to a particular environment. Advanced studies have reported that surface charged residues impact enzymatic conformational stability through electrostatic interactions, hydrogen bonds, and interactions with water molecules from the surrounding environment (Warden et al., 2015; Pedersen et al., 2019). In general, the conformational stability is related to the tolerance of extreme pH values, high salinity, metal ions, and elevated temperatures (Sokalingam et al., 2012; Raghunathan et al., 2013; Kumar S. et al., 2019). In alkaline conditions, the stability of the enzyme is typically characterized by a decreased number of acidic residues and an increased number of arginine residues (Collins et al., 2005; Mamo et al., 2009). Conversely, acidstable enzymes are characterized by a high concentration of acidic residues on the protein surface (Fushinobu et al., 1998).

To better understand key factors associated with acid-tolerance and alkali-resistance in enzymes and to develop novel biocatalysts for extreme reaction conditions, we designed and conducted experiments to create pH tolerant xylanases by mutating residues on the enzyme surface of thermostable *Tl*XynA from *T. lanuginosus* screened from biomass composting (Zhang et al., 2015). Herein, we altered 10-residues by creating point mutations on the surface of *Tl*XynA and successfully improved its ability to adapt to extreme pH levels. This study confirmed the role of charged surface residues and developed mutants with enhanced stabilities that are of great significance for both understanding enzymatic functions and using in industrial processes.

## Materials and Methods

### Phylogenetic Analysis

All protein sequences and structures of GH11 xylanases were downloaded from the Carbohydrate-Active enZYmes database (CAZy)^1^ and Protein Data Bank (PDB)^2^. Multiple sequence alignments were performed with the CLUSTAL algorithm using MEGA5 (Larkin et al., 2007). A neighbor-joining phylogenetic tree was constructed with bootstrap test based on 24 members with known structures, and further optimized by iTOL (Letunic and Bork, 2016). Surface charged amino acids were identified using MetaPocket2.0 (Huang, 2009; Zhang et al., 2011), and the sequence profile of charged surface residues of the GH11 family was created using WebLogo (Schneider and Stephens, 1990; Crooks et al., 2004). Sequence alignment images were produced using ESPript (Robert and Gouet, 2014). Surface charges were displayed using the “vacuum electrostatics” selection in PyMOL.

### Strains, Medium, and Plasmids

*Escherichia coli* DH5α and BL21(DE3) (Dingguo, Beijing, China) were used for molecular cloning and secretory expression of xylanases, respectively. All *E. coli* strains were grown in LB medium. The pET28a plasmid (Thermo Fisher Scientific, Waltham, MA, United States) was used as the expression vector and then assembled with the *Tl*XynA gene (NCBI accession number: AAB94633) to produce the pET28a-*Tl*XynA plasmid for subsequent mutagenesis experiments.

### Site-Directed Mutagenesis

The *Tl*XynA gene (NCBI accession number: AAB94633) was used as the wild-type (WT) enzyme, and site-directed mutagenesis was performed using a PCR-based method (Weiner et al., 1994). PCR was performed (S1000 Thermal Cycler; Bio-Rad, Hercules, CA, United States) using PrimeSTAR Max Premix (TaKaRa, Shiga, Japan). The sequences of primers used for mutagenesis are listed in Supplementary Table S1. After digesting with *Dpn*I (Thermo Fisher Scientific, Waltham, MA, United States) and purifying the fragment, the PCR product was transformed into competent *E. coli* DH5α cells and selected using LB-kanamycin plates. Plasmid recovery was conducted using the GV-Plasmid DNA Mini Extraction Kit (Dingguo, Beijing, China) and the plasmids were sequenced (Tsingke, Qingdao, China) to verify the mutations.

### Protein Expression and Purification

The verified plasmids were transformed into *E. coli* BL21 (DE3) cells, which was then cultured in LB medium supplemented with 50 μg/mL kanamycin (Sangon Biotech, Shanghai, China) at 37°C until OD_600_ = 0.6–0.8, followed by inducing expressing using 0.5 mM isopropyl-β-D-1-thiogalactopyranoside (IPTG; Sangon Biotech, Shanghai, China) for 20 h at 20°C. *E. coli* BL21 (DE3) cells were harvested by centrifugation and resuspended in lysis buffer (50 mM NaH_2_PO_4_ and 300 mM NaCl at pH 8.0). After ultrasonic fragmentation, the His-tagged proteins were purified using a Ni Sepharose 6 Fast Flow column (GE Healthcare, Uppsala, Sweden). The eluent was replaced with PC buffer (20 mM sodium phosphate and 10 mM citrate at pH 6.0) and then filtered using a 3 kDa cutoff membrane (Millipore, Billerica, MA, United States) at 4°C. The protein obtained was analyzed by sodium dodecyl sulfate-polyacrylamide gel electrophoresis. Protein concentrations were determined using the Bradford method (Bradford, 1976).

### Circular Dichroism (CD) Experiments

A CD spectrophotometer J-600 (Jasco, Tokyo, Japan) was used to measure the structural changes of the WT and mutant enzymes in PC buffer (50 mM sodium phosphate and 20 mM citrate at pH 6.0) at 20°C, which is usually used to determine the secondary structures of proteins (Whitmore and Wallace, 2008). The concentration of proteins was ~0.08 mg/mL. The scan rate was set at 20 nm/min. Samples were scanned at wavelengths of 190 nm to 250 nm with three replicate experiments.

### Differential Scanning Calorimetry (DSC) Measurements

The melting temperature (T_m_) analysis of each protein was performed using a MicroCal VP-Capillary differential scanning calorimeter (MicroCal, Northampton, MA, United States); this technique is usually used to determine the thermostability of proteins (Durowoju et al., 2017). The enzymes were dissolved in PC buffer to a final concentration of 0.02 mM. Both the protein samples and buffer were degassed, and 400 μL of the protein samples and buffer were added into DSC sample and reference cells, respectively. DSC scans were performed at temperatures ranging from 20 to 100°C with a heating rate of 1°C/min.

### Activity Measurements

The substrate was 1% beechwood xylan (Megazyme, Wicklow, Republic of Ireland). Enzymes were dissolved in PC buffer (pH 6.0) at a final concentration of 0.5 μg/mL. The substrates were dissolved either in PC buffer with different pH values ranging from pH 3.0–8.0 or at different concentrations of NaCl. After reacting at 65°C for 10 min, the amount of reducing sugar was determined using the dinitrosalicylic acid reagent method (Miller, 1959), and the absorbance was measured at 540 nm using a SpectraMax M5 Microplate Reader (Bihe, Shanghai, China). One unit of xylanase activity (U) corresponded to the amount of enzyme that released 1 μmol of reducing sugar equivalent from xylan per minute.

### Kinetic Analysis

Kinetic analyses of the catalytic reactions were performed under standard conditions using purified enzymes (100 μL) and various concentrations of xylan substrates (500 μL) for 3 min at 65°C, and then 400 μL DNS solution was added to terminate the reaction. The kinetic parameters including the turnover rate (*k*_cat_), Michaelis constant (*K*_m_), and catalytic efficiency (*k*_cat_/*K*_m_) were non-linearly fitted based on the Michaelis-Menten equation using Prism 5.0 (GraphPad, San Diego, CA, United States) (Motulsky, 2007).

## Results

### Differences in the Surface Charged Residues of GH11 Xylanases

**FIGURE 1:**
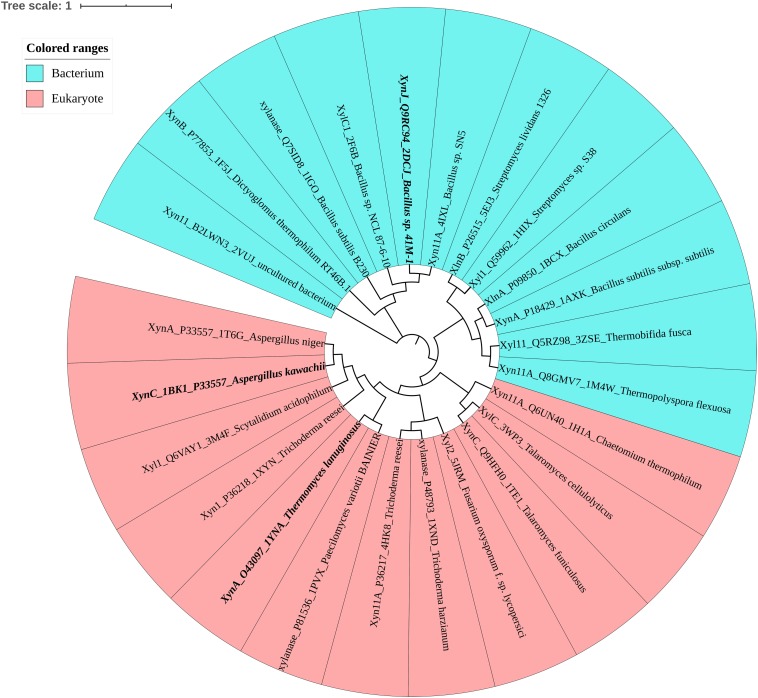
Phylogenetic tree of the GH11 family, in which bacteria are shown in blue and eukaryotes are shown in pink. Mesophilic *Tl*XynA (PDB ID: 1YNA) from *T. lanuginosus*, acid-resistant XynC (PDB ID: 1BK1) from *A. kawachii*, and alkali-resistant XynJ (PDB ID: 2DCJ) secreted by *Bacillus sp.* 41M1 are bold. The text includes the enzyme name, Uniprot ID, PDB ID, and the source of the microbe.

To investigate the determinants associated with ion resistance of GH11 xylanases, the phylogenetic tree and amino acid composition were analyzed. Figure 1 shows that based on the protein sequence similarities, GH11 xylanases were divided into two separate groups: bacterium and eukaryote. Among these, three xylanases were selected for further study (Table 1). XynJ (PDB ID: 2DCJ) is secreted by *Bacillus* sp. 41M1 with a pH_opt_ of 9.0 and an activity that is at least 60% of the optimal activity at a pH range from 5.0 to 9.5 (Nakamura et al., 1993). XynC (PDB ID: 1BK1) is a halotolerant and acidic xylanase from *A. kawachii* with an optimum pH of 2.0 (Fushinobu et al., 1998). *Tl*XynA (PDB ID: 1YNA) is a thermostable enzyme with an optimum of pH 6.0, and has a closer evolutionary relationship with *Ak*XynC than with *Bs*XynJ (Figure 1).

**TABLE 1:** Characteristics of the studied GH11 xylanases.

**Enzyme**	**Organism**	**PDB ID**	**GenBank**	**pH_*opt*_**	**Others**
*Tl*XynA	*Thermomyces lanuginosus*	1YNA	AAB94633.1	6.0	Thermostability
*Bs*XynJ	*Bacillus sp.* 41M-1	2DCJ	BAA82316.1	9.0	60% optimal activity at pH 5.0–9.5
*Ak*XynC	*Aspergillus kawachii*	1BK1	AAC60542.1	2.0	Halotolerance

**FIGURE 2:**
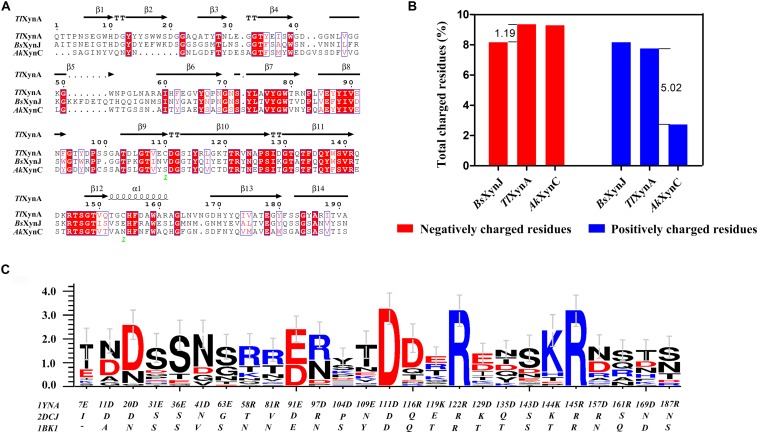
Sequence alignment and charged residue composition analysis of *Tl*XynA (PDB ID: 1YNA), *Bs*XynJ (PDB ID: 2DCJ), and *Ak*XynC (PDB ID: 1BK1). **(A)** Sequence alignment map of *Tl*XynA, *Bs*XynJ, and *Ak*XynC. Strictly conserved residues are highlighted by a red background, and conservatively substituted residues are boxed. The secondary structure of *Tl*XynA (PDB ID: 1YNA) is shown above the aligned sequences. The green numbers represent the location of the disulfide bond. **(B)** Total charged residues (%) in the three xylanases: *Tl*XynA, *Bs*XynJ, and *Ak*XynC. The blue rectangles represent the composition ratio of the positively charged amino acids, and the red rectangles represent the composition ratio of the negatively charged amino acids. **(C)** Sequence profile of charged surface residues in the GH11 family using *Tl*XynA (PDB ID: 1YNA) as a template.

Sequence alignment was performed to analyze the three enzymes (Figure 2A). The sequence similarity was 44.44% between *Tl*XynA and *Bs*XynJ and 41.75% between *Tl*XynA and *Ak*XynC. The total amino acid compositions of the catalytic domains for the 24 xylanases are provided in Supplementary Table S2. The three enzymes had different proportions of amino acids, especially charged amino acids. Figure 2B shows that the aciduric and halotolerant xylanase, *Ak*XynC, contained very few positively charged amino acids (2.73%), while there were 7.75% positively charged residues in *Tl*XynA and 8.16% in *Bs*XynJ. The alkaline active xylanase *Bs*XynJ has the lowest percent (8.16%) of negatively charged residues (Asp and Glu) than *Tl*XynA and *Ak*XynC. Furthermore, the electrostatic features of the surface of these three enzyme molecules were obviously different (Supplementary Figure S1). Next, the sequence profile of surface polar residues of the GH11 family enzymes was constructed with *Tl*XynA as a template. Figure 2C shows nine positively and 17 negatively charged amino acids. Three surface charged residues, Arg122, Lys144, and Asp111, are completely conserved, implying that they may play key roles in the enzymatic function. However, most charged residues were variable, suggesting that these sites could be potential mutant sites for changing the enzymatic function. Differences in positively and negatively charged surface amino acids would, therefore, be expected to give rise to differences in the enzymatic function of GH11 xylanase.

Based on the above analysis, four positively charged surface residues from *Tl*XynA, Arg116, Lys119, Arg161, and Arg187, were chosen to be mutated. According to the sequence of *Ak*XynC, these were mutated to non-polar residues glutamine, threonine, glutamine, and serine, respectively (Figure 2C). In addition, six non-conservative negatively charged surface residues, Glu31, Glu36, Glu63, Glu109, Asp143, and Asp157, were mutated to uncharged amino acids (either asparagine or serine) to reduce the ratio of negatively charged residues (Figure 2C). The mutant list is shown in Table 2, and the mutation sites are shown in the structure of *Tl*XynA (Supplementary Figure S2).

**TABLE 2:** The mutants listed in this study.

**Name**	**Enzyme**
WT	*Tl*XynA
P1^a^	R116Q/R161Q
P2^a^	R116Q/K119T
P3^a^	R116Q/R161Q/R187S
P4^a^	R116Q/R161Q/R187S/K119T
N1^b^	E63N/E109N
N2^b^	E31S/E63N/E109N
N3^b^	E31S/E63N/E109N/D143S
N4^b^	E31S/E36S/E63N/E109N/D143S
N5^b^	E31S/E36S/E63N/E109N/D143S/D157N

*^a^The P series mutants represent positively charge residues mutations.*

*^*b*^The N series mutants represent negatively charge residues mutations.*

### The Impacts of Positively Charged Residue Mutations on the Enzymatic Properties

**FIGURE 3:**
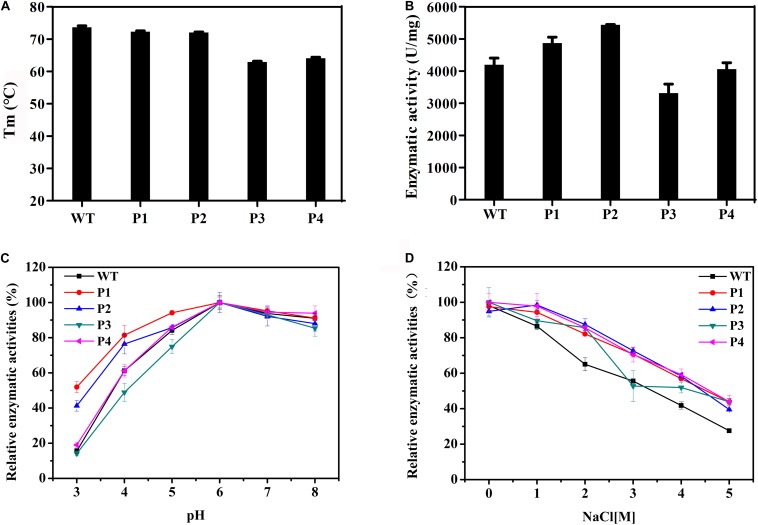
Biochemical properties of WT and mutants P1, P2, P3, and P4. **(A)** The thermostabilities of the WT and these variants determined by differential scanning calorimetry. **(B)** The enzymatic activities of WT and these variants performed using beechwood xylan at 65°C. **(C)** The relationship between the pH and activity for the WT and variant enzymes. **(D)** Determination of activities of WT and these variants using a gradient NaCl solution.

WT and mutant enzymes were successfully heterologous expressed and purified (Supplementary Figure S3). The secondary structures of the WT and mutant enzymes did not change (Supplementary Figure S4) and there were no much changes in 3D structure because of amino acid substitutions (RMSD = 0.054). The thermostability of the purified proteins was determined by DSC. As shown in Figure 3A, with the exceptions of P3 and P4, the thermostability of these enzymes was not significantly affected. Compared with the WT enzyme, the T_m_ values of two mutants P3 and P4 decreased by ∼10°C. It is possible that these mutations disrupted the surface interaction network. Figure 3B shows that the activities of the P1 and P2 mutants increased by 16 and 30%, respectively, while the catalytic activities of the P3 and P4 mutants slightly decreased. The *K*_m_ values for the P1 and P2 mutants were significantly reduced (Table 3), indicating that the binding affinity was higher in these mutants, while the P3 and P4 mutants displayed few changes in the binding affinity. Except for the P4 variant, the turnover rate (*k*_cat_) of these mutants was lower than that of the WT enzyme. Finally, the catalytic efficiencies (*k*_cat_/*K*_m_) of the P1 and P2 mutants were higher than that of WT, while the P3 and P4 mutants had lower catalytic efficiencies. The T_m_ values and specific activities of the WT and mutant enzymes are shown in Supplementary Table S3.

**TABLE 3:** Kinetic parameters of the WT and variants (P1, P2, P3, and P4).

**Enzyme**	***K*_m_ (g/L)**	***k*_cat_ (g/L)**	***k*_cat_/*K*_m_ (L/s/g)**
WT	4.38	7,025	1,604
P1	3.39	5,925	1,748
P2	3.15	5,842	1,854
P3	4.49	5,715	1,273
P4	4.75	7,071	1,489

The catalytic activities of these enzymes were determined between pH 3.0 and 8.0 (Figure 3C). The results showed that the WT and variant enzymes had a similar optimal pH of ∼6.0. With the exception of the P4 mutant, the activities of the other mutants were not consistent with that of the WT (Figure 3C). Importantly, at pH 3.0, P1 and P2 presented 50 and 40% of their maximum activity, respectively, when the WT and other two mutant enzymes were almost completely inactivated. These results indicated that the P1 and P2 mutants were more resistant to acidic environments, while the P3 mutant had a negative effect on the enzymatic catalysis. The enzyme activities of the WT and mutant enzymes at different salt concentrations were also measured. Figure 3D shows that the relative enzyme activities of all four variants were higher than that of the WT when NaCl was added to the reaction system. The maximum enzyme activities of these mutants were 1.6 times that of the WT in 5 M NaCl. These results indicated that these four mutants had enhanced tolerance to high concentrations of salt ions.

### The Impacts of Negatively Charged Residue Mutations on the Enzymatic Properties

**FIGURE 4:**
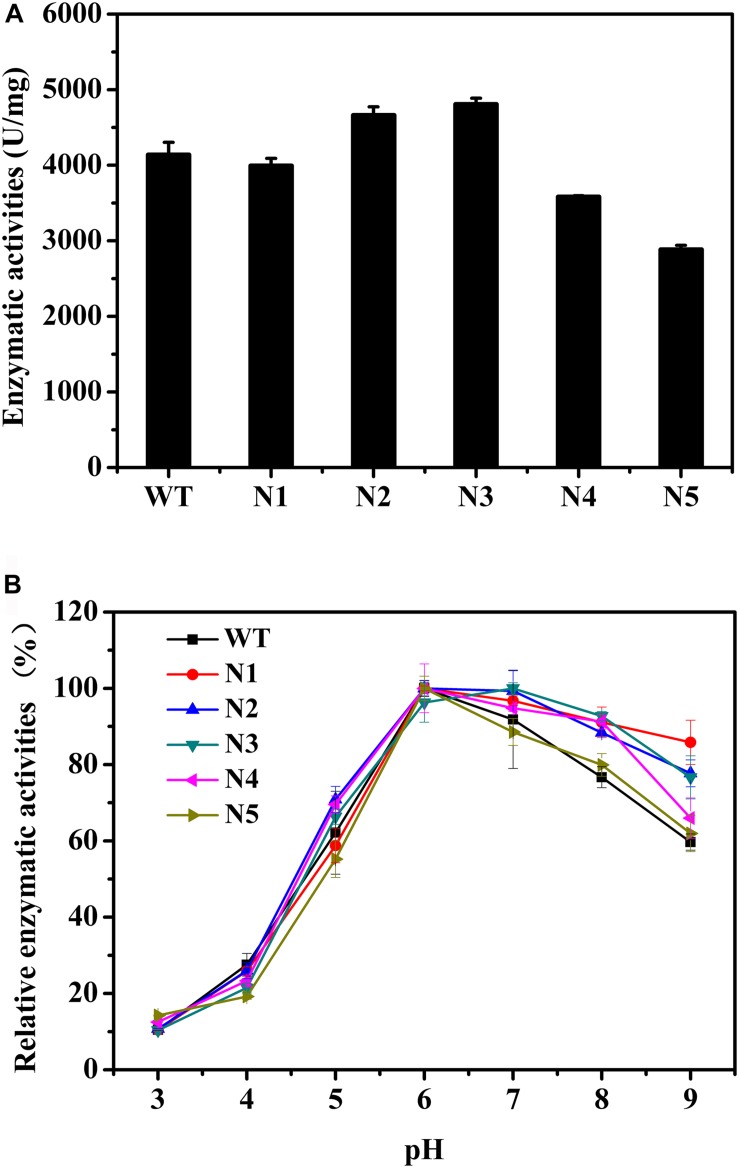
Biochemical properties of WT and mutants N1, N2, N3, N4, and N5. **(A)** Enzymatic activities of WT and these mutants determined using beechwood xylan at 65°C. **(B)** Relationship between pH and enzymatic activity for WT and these variants.

We also mutated a series of negatively charged amino acids, and these corresponding mutant enzymes were also successfully heterologous expressed and purified (Supplementary Figures S3, S4). The thermostabilities of the purified proteins were determined by DSC (Table 4). Compared with the WT, the thermostabilities of N1, N2, and N3 barely changed, while those of N4 and N5 slightly decreased. The T_m_ values of N4 and N5 decreased by ∼4°C. Figure 4A shows that the enzyme activities of the N2 and N3 variants increased by 13 and 16%, respectively. The main reason for the increase in the enzyme activities was that the turnover rate (*k*_cat_) of these two mutants was higher than that of the WT, although the *K*_m_ values increased. Finally, the catalytic efficiencies (*k*_cat_/*K*_m_) of the N2 and N3 mutants were higher than that of WT (Table 4). The catalytic activities of the N1 and N4 variants were slightly lower than that of the WT, and the N5 mutant had the lowest enzymatic activity (Figure 4A). The specific activities of the WT and mutant enzymes are shown in Supplementary Table S4.

**TABLE 4:** Kinetic parameters of WT and variants (N1, N2, N3, N4, and N5).

**Enzyme**	**T_m_ (°C)**	***K*_m_ (g/L)**	***k*_cat_ (g/L)**	***k*_cat_/*K*_m_ (L/s/g)**
WT	73.65	4.38	7,025	1,604
N1	73.5	5.56	8,727	1,570
N2	73.25	6.27	13,237	2,111
N3	73.35	6.16	13,106	2,127
N4	69.85	5.69	7,998	1,406
N5	69.5	6.03	5,806	963

The catalytic activities of these recombinant enzymes were determined between pH 3.0 and 9.0. Figure 4B shows that the optimal pH of these mutants was the same as that of the WT. Except for the N5 mutant, as the pH increased, the activity profiles of other mutants decreased more slowly than that of the WT. In addition, at pH 9.0, N1, N2, N3, and N4 presented 86, 78, 77, and 66% of their maximum activities, respectively, while the WT and N5 mutant retained ∼60% of their enzyme activities (Figure 4B). Taken together, the observations indicated that these mutants, with the exception of the N5 mutant, were more tolerant to alkaline environments.

## Discussion

In nature, a neutral environment is the most common catalytic condition for most enzymes, and these conditions include a moderate temperature, pH, and salinity range. Harsh conditions usually lead to the rapid denaturation of enzyme molecules (Collins et al., 2005). Enzymes secreted by extremophiles under extreme environments evolved adaptive activity and stability characteristics (Demirjian et al., 2001). However, in the course of evolution, a significant number of beneficial mutations occurred, resulting in the ability to adapt to their respective living conditions. These interesting modifications can be employed to rationally design enzymes with improve tolerance to extreme conditions (Camps et al., 2007; Zanphorlin et al., 2019). Currently, strategic utilization of protein engineering methods has endowed better enzymatic properties to suit a wide range of applications in various industries (Kumar et al., 2016, 2018; Basu et al., 2018). Moreover, significant progress has been made in engineering the surface charge of proteins. Modifying surface charges has been increasingly used to enhance enzyme properties, including thermostability (Bipin et al., 2015), IL tolerance (Nordwald et al., 2014), ligin tolerance (Whitehead et al., 2017), and halophilicity (Warden et al., 2015). Although xylanases of the GH11 family have very similar three-dimensional structures, the optimum pH values are highly variable within this family and range between pH 2.0 and 11.0 (Paes et al., 2012). The characteristics of acidstable and alklistable xylanases can provide inspiration for improving the pH tolerance of xylanases. To the best of our knowledge, a wide variety of comparative investigations of the functional residues in the active site of thermophilic protein with their mesophilic counterparts have been experimentally and theoretically studied (Cheng et al., 2014; Harris et al., 2018; Wu et al., 2018). Therefore, in this study, the charged surface residues of naturally existing acidstable, alklistable and neutral enzymes were compared and analyzed, and amino acid residues that may affect the pH tolerance were identified and mutated.

Although it is difficult to know the role of each amino acid in the overall property of an enzyme, a comparison of homologous proteins may confirm some significant trends in amino acid frequencies that could be correlated to the adaptations adopted by proteins to exist in extreme environments (Fushinobu et al., 1998; Mamo et al., 2009). The analysis of amino acid frequencies showed that the percentages of charged amino acids were significantly different (Figure 2B). Acidic and halophilic enzymes contain the least positively charged amino acids and alkalic enzymes possess fewer negatively charged amino acids. The nature of a protein surface is of vital importance in adapting to a particular environment (Mamo et al., 2009; Warden et al., 2015). Three xylanases adapted to different pH environments also showed changes on their surfaces (Supplementary Figure S1). More negatively charged residues were found on the surface of an extremely acidophilic xylanase from *A. kawachii* (Fushinobu et al., 1998). Furthermore, a highly active alkaline protease (Shirai et al., 1997) and the alkali-stable xylanase *Bs*XynJ contain excess positively charged residues on the surface (Supplementary Figure S1B). Halophilic enzymes have more negative charges on their surface (Warden et al., 2015). These observations suggest that changes in the ratio of negatively and positively charged residues on the protein surface may contribute to the adaptation of GH11 xylanases to extreme pH levels. Figure 3C shows that increasing the negative/positive ratio of surface residues enhanced the acid resistance of an enzyme, whereas reducing the negative/positive ratio of surface residues improved the alkali-resistance (Figure 4B). Several studies have shown that increasing the number of arginines on the protein surface was involved in the adaptation of enzymes to highly alkaline pH levels (Turunen et al., 2002; Kumar et al., 2016). However, a reduction in the frequency of positively charged surface residues, such as Arg and Lys, is likely to contribute to the high tolerance of the enzymes at a low pH.

Xylanase from *T. lanuginosus* is a thermostable enzyme with an optimum temperature of 65°C (Kumar et al., 2017), and as a commercial enzyme, it is widely used in various fields, such as xylan degradation (Palaniappan et al., 2017), oligosaccharide production (Van Munster et al., 2017), viscosity measurement (Hernández et al., 2008), and the commercial production of hydroenzymatic enzymes from corn germ (Moreau et al., 2004). In this study, *Tl*XynA was obtained from *T. lanuginosus*, an isolate from maize straw composts (Damaso et al., 2003; Zhang et al., 2015; Shi et al., 2019). The enzymatic activity of *Tl*XynA (≥4,000 U/mg) was 1,000-fold than commercial xylanase from *T. lanuginosus* (≥2,500 U/g, recombinant, Sigma Aldrich product). On this basis, the application range of xylanase can be widened by increasing the acid or alkali resistance. In the field of oligosaccharide production, finger millet seed coat was degraded with commercial xylanase from *T. lanuginosus* at pH 4.8 (Palaniappan et al., 2017). P1 and P2 mutants show better ability of degrading pretreated biomass substrates because of maintaining high enzyme activity at low pH (pH 3.0–6.0) and high salt condition (5 M NaCl). Moreover, the xylanase (221.56 U/mg) obtained from *T. lanuginosus* VAPS24 was capable of bleaching mixed hardwood pulp (Kumar and Shukla, 2018; Kumar V. et al., 2019). N2 and N3 variants maintained around 80% of WT enzymatic activity under pH 9.0 conditions could be widely used in bleaching industry. In addition, increased pH tolerance can reduce the loss of enzyme activity during the production of enzyme preparations. In fact, some xylanases isolated from the extreme environment showed better properties compared with our mutants. For instance, the GH11 xylanase rMxyl identified from compost-soil by metagenomic approach (Verma et al., 2013) and GH10 endoxylanase of *Bacillus halodurans* isolated from the paper mill effluents (Kumar and Satyanarayana, 2011) exhibited activity over a broad range of pH and temperature with optima at pH 9.0 and 80°C. Therefore, further attempts in improving the pH-tolerance of *Tl*XynA are needed. In addition, the design strategy could be also utilized in further enhancing and extending the properties and their application range of those extreme enzymes.

The rational design strategy used in this paper is effective in design of pH tolerance of enzymes. First, the corresponding structural domain was selected according to the performance of the protein, then the mutation sites and direction of mutation were identified through a natural evolution analysis, and finally, experimental verifications were carried out. The T_m_ value showed that the surface charged residues Arg187 and Glu36 may affect the thermal stability of the enzyme (Figure 3A and Table 4). Local structural analysis showed that hydrogen bond interactions existed with the surrounding amino acids (Supplementary Figure S5). Changes in the amino acid protonation state often altered electrostatic interactions and spatial arrangements in the protein structure (Horng et al., 2005; Mazzini et al., 2007). This result suggests that surface residues that are not involved in significant interactions may be mutated. The effective release of the product may be due to the reduction in charged surface residues, which may produce non-specific binding; however, the formation of non-specific binding needs to be supported by further structural studies.

## Conclusion

In conclusion, comparative analysis of three different xylanases, *Tl*XynA, *Bs*XynJ, and *Ak*XynC provided possible mechanisms of enzymatic activity and stability under various pH conditions. More importantly, four mutants with better properties than that of the WT enzyme have been evolved. This method to rationally design charged surface residues has paved a new way for understanding and engineering acid and alkali stable xylanases and for characterizing other enzymes. Further, continued research to optimize the properties of thermozyme will help in designing tailor made enzyme for specific application.

## Data Availability Statement

All datasets generated for this study are included in the article/Supplementary Material.

## Author Contributions

XW designed the experiments and wrote the manuscript. QZ, LZ, and SL performed the experiments and analyzed the data. GC, HZ, and LW conceived the study design and edited the paper. All authors read and approved the final manuscript.

## Conflict of Interest

The authors declare that the research was conducted in the absence of any commercial or financial relationships that could be construed as a potential conflict of interest.

## Footnotes

1 http://www.cazy.org/Glycoside-Hydrolases.html

2 http://www.rcsb.org
