# Supplementary material for: Insights Into the Role of Exposed Surface Charged Residues in the Alkali-Tolerance of GH11 Xylanase
Source: Front Microbiol. 2020 May 8;11:872. doi: 10.3389/fmicb.2020.00872 (PMC7225583; doi:10.3389/fmicb.2020.00872)
Supplement: Supplementary file 1 [file Data_Sheet_1.docx]

Supplementary Material

**Table S1 The primers sequences used in this article.**

| Name | Sequence (5’ to 3’) |
| --- | --- |
| R116Q-sense | GGTAGCATCTATCAACTCGGCAAGA |
| R116Q-antisense | TCTTGCCGAGTTGATAGATGCTACC |
| K119T-sense | ATCTATCAACTCGGCACGACCACTA |
| K119T-antisense | TAGTGGTCGTGCCGAGTTGATAGAT |
| R161Q-sense | CCTGGGCTCAAGCTGGTTTGAA |
| R161Q-antisense | ACATTCAAACCAGCTTGAGCCCAG |
| R187S-sense | CTATGCTAGCATCACCGTTGCTGAC |
| R187S-antisense | GTCAGCAACGGTGATGCTAGCATAG |
| E31S-sense | ACACCAACCTGAGTGGCGGCACCTA |
| E31S-antisense | AGGTGCCGCCACTCAGGTTGGTGTA |
| E36S-sense | AAGGCGGCACCTACAGTATCAGCTG |
| E36S-antisense | AGCTGATACTGTAGGTGCCGCCTTC |
| E63N-sense | AGCCATCCACTTTAATGGTGTTTAC |
| E63N-antisense | GGTAAACACCATTAAAGTGGATGGC |
| E109N-sense | TCTAGGAACTGTCAATTGCGACGGT |
| E109N-antisense | GCTACCGTCGCAATTGACAGTTCCT |
| D143S-sense | TACTGGTCGGTCCGCCAGAGTAAGC |
| D143S-antisense | GCTTACTCTGGCGGACCGACCAGTA |
| D157N-sense | AGACGGGCTGCCACTTCAATGCCT |
| D157N-antisense | AGGCATTGAAGTGGCAGCCCGTCT |

**Table S2 Total amino acids composition (%) of family 11 xylanases.**

| **Amino acid** | **2VUJ** | **1F5J** | **1IGO** | **2F6B** | **2DCJ** | **4IXL** | **5EJ3** | **1HIX** | **1BCX** | **1AXK** | **3ZSE** | **1M4W** | **1H1A** | **3WP3** | **1TE1** | **5JRM** | **1XND** | **4HK8** | **1PVX** | **1YNA** | **1XYN** | **3M4F** | **1BK1** | **1T6G** |
| --- | --- | --- | --- | --- | --- | --- | --- | --- | --- | --- | --- | --- | --- | --- | --- | --- | --- | --- | --- | --- | --- | --- | --- | --- |
| **Arg** | **4.21** | **5.18** | **2.58** | **3.85** | **3.06** | **2.75** | **4.81** | **3.72** | **3.78** | **3.78** | **5.35** | **5.35** | **5.26** | **2.15** | **1.59** | **3.43** | **3.16** | **3.16** | **3.14** | **4.19** | **1.69** | **2.65** | **1.64** | **1.64** |
| **Lys** | **4.21** | **2.07** | **6.19** | **4.95** | **3.57** | **2.75** | **2.67** | **2.13** | **2.7** | **2.7** | **1.60** | **1.6** | **1.05** | **1.61** | **1.59** | **4.00** | **2.11** | **2.11** | **1.05** | **1.57** | **0.56** | **0.53** | **0** | **0.55** |
| **His** | **1.05** | **0.52** | **1.55** | **1.65** | **1.53** | **3.30** | **0.53** | **0.53** | **1.08** | **1.08** | **2.67** | **1.6** | **1.58** | **0.54** | **0.53** | **1.71** | **2.11** | **1.58** | **2.09** | **2.09** | **1.39** | **1.06** | **1.09** | **1.09** |
| **Asp** | **3.68** | **3.63** | **4.12** | **3.85** | **3.57** | **6.59** | **3.21** | **2.66** | **3.78** | **3.78** | **3.74** | **3.21** | **3.16** | **3.23** | **3.17** | **2.86** | **2.11** | **2.11** | **4.71** | **5.16** | **2.81** | **5.29** | **4.92** | **4.92** |
| **Glu** | **1.05** | **2.07** | **3.61** | **3.85** | **4.59** | **6.59** | **2.67** | **1.6** | **1.08** | **1.08** | **3.21** | **2.67** | **2.63** | **5.38** | **5.29** | **2.29** | **2.11** | **2.11** | **2.62** | **4.19** | **2.81** | **4.76** | **4.37** | **4.37** |
| **Asn** | **8.42** | **8.81** | **7.73** | **9.34** | **8.16** | **3.85** | **8.02** | **8.51** | **9.73** | **9.73** | **5.35** | **5.88** | **8.42** | **7.53** | **7.41** | **10.29** | **10** | **10.53** | **7.33** | **6.28** | **10.11** | **6.35** | **6.56** | **6.01** |
| **Gln** | **4.21** | **5.18** | **3.61** | **4.4** | **4.59** | **4.95** | **3.74** | **2.66** | **2.70** | **2.70** | **1.6** | **4.28** | **3.68** | **3.23** | **3.17** | **4.0** | **3.16** | **5.26** | **3.66** | **4.19** | **6.18** | **3.7** | **3.28** | **3.28** |
| **Gly** | **14.74** | **9.84** | **11.34** | **12.09** | **12.24** | **12.09** | **14.44** | **14.89** | **13.51** | **13.51** | **13.9** | **12.83** | **14.21** | **12.9** | **13.23** | **14.86** | **14.21** | **14.21** | **15.71** | **14.66** | **12.36** | **13.23** | **10.38** | **10.38** |
| **Pro** | **3.16** | **2.59** | **3.09** | **3.30** | **3.06** | **3.30** | **2.67** | **2.66** | **3.24** | **3.24** | **3.21** | **2.67** | **2.63** | **2.15** | **2.65** | **3.43** | **3.16** | **3.68** | **3.14** | **3.14** | **3.37** | **2.65** | **1.64** | **1.64** |
| **Thr** | **10** | **14.51** | **13.40** | **7.69** | **10.71** | **10.44** | **12.30** | **12.23** | **13.51** | **12.97** | **14.97** | **14.97** | **11.58** | **13.98** | **14.81** | **6.86** | **8.95** | **8.42** | **10.99** | **9.42** | **10.11** | **11.11** | **10.93** | **10.93** |
| **Tyr** | **7.37** | **7.25** | **6.7** | **6.59** | **7.14** | **6.59** | **9.09** | **9.57** | **8.11** | **8.11** | **9.09** | **8.56** | **9.47** | **10.22** | **9.52** | **9.14** | **9.47** | **8.95** | **8.9** | **8.9** | **10./5.62** | **8.47** | **9.29** | **9.29** |
| **Ser** | **12.63** | **8.81** | **9.28** | **7.69** | **10.71** | **8.24** | **10.70** | **11.70** | **9.73** | **10.27** | **9.09** | **10.70** | **8.95** | **1075** | **11.64** | **12** | **12.63** | **11.58** | **11.52** | **6.81** | **12.92** | **11.64** | **15.30** | **15.30** |
| **Trp** | **4.74** | **4.15** | **3.09** | **3.85** | **3.57** | **4.4** | **3.74** | **9.57** | **5.95** | **5.95** | **3.74** | **4.28** | **3.68** | **3.76** | **3.17** | **3.43** | **3.16** | **3.16** | **4.19** | **4.19** | **3.37** | **2.65** | **2.73** | **2.73** |
| **Ala** | **3.68** | **4.15** | **4.12** | **4.4** | **3.57** | **4.4** | **4.28** | **5.85** | **4.86** | **4.86** | **5.88** | **4.81** | **5.79** | **4.30** | **4.23** | **3.43** | **4.74** | **3.68** | **4.71** | **6.81** | **5.06** | **7.94** | **7.65** | **7.65** |
| **Val** | **5.79** | **5.18** | **4.12** | **6.59** | **5.16** | **6.04** | **6.42** | **6.38** | **7.57** | **7.57** | **4.81** | **4.28** | **6.84** | **6.99** | **6.88** | **7.43** | **6.84** | **7.37** | **5.76** | **6.28** | **10.11** | **7.94** | **8.2** | **8.2** |
| **Phe** | **3.68** | **3.11** | **3.61** | **3.85** | **3.57** | **3.85** | **3.21** | **3.19** | **2.16** | **2.16** | **3.21** | **3.74** | **2.63** | **3.23** | **2.65** | **3.43** | **3.68** | **4.21** | **2.62** | **2.62** | **3.37** | **3.7** | **4.92** | **4.92** |
| **Ile** | **4.21** | **5.7** | **5.15** | **4.95** | **5.1** | **4.40** | **2.67** | **2.66** | **3.24** | **3.24** | **2.67** | **4.28** | **4.21** | **2.15** | **3.17** | **3.43** | **5.26** | **4.74** | **3.14** | **3.66** | **3.37** | **2.12** | **2.73** | **2.73** |
| **Leu** | **4.21** | **5.18** | **4.12** | **3.85** | **3.06** | **3.30** | **2.14** | **2.66** | **2.16** | **2.16** | **3.21** | **2.67** | **3.16** | **4.3** | **3.7** | **2.86** | **2.63** | **2.63** | **3.66** | **4.19** | **3.37** | **2.12** | **2.19** | **2.19** |
| **Cys** | **1.05** | **1.55** | **0.52** | **0.55** | **0** | **0** | **0** | **0.53** | **0** | **0** | **0** | **0** | **0** | **0.54** | **0.53** | **0** | **0** | **0** | **1.05** | **1.05** | **0** | **1.59** | **1.09** | **1.09** |
| **Met** | **0.53** | **0.52** | **2.06** | **2.75** | **2.55** | **2.2** | **2.67** | **2.13** | **1.08** | **1.08** | **2.67** | **1.6** | **1.05** | **1.08** | **1.06** | **1.14** | **0.53** | **0.53** | **0** | **0** | **1.12** | **0.53** | **1.09** | **1.09** |
| **Number of total charged residues ratio (-ve/+ve)** | | | | | | | | | | | | | | | | | | | | | | | | |
|  | **0.50** | **0.73** | **0.75** | **0.74** | **1.00** | **1.50** | **0.73** | **0.67** | **0.64** | **0.64** | **0.72** | **0.69** | **0.73** | **2** | **2.29** | **0.56** | **0.57** | **0.62** | **1.17** | **1.27** | **1.43** | **2.38** | **3.4** | **2.83** |

The positively charged amino acids Arg, Lys and His and their proportions are shown in blue, and the negatively charged amino acids Asp and Glu and their proportions are shown in red.

**Table S3 Tm and specific activities of WT and variants (P1, P2, P3 and P4)**

| Enzyme | Tm  (^o^C) | Specific activity (U/mg) |
| --- | --- | --- |
| WT | 73.65 | 4196.25±209.22 |
| P1 | 72.30 | 4873.18±183.25 |
| P2 | 72.10 | 5444.09±8.82 |
| P3 | 62.90 | 3319.29±275.88 |
| P4 | 64.05 | 4061.97±198.09 |

**Table S4 Specific activities of WT and variants (N1, N2, N3, N4 and N5)**

| Enzyme | Specific activity (U/mg) |
| --- | --- |
| WT | 4183.90±164.94 |
| N1 | 3993.79±95.90 |
| N2 | 4662.76±110.49 |
| N3 | 4807.57±79.87 |
| N4 | 3581.53±15.48 |
| N5 | 2884.42±54.29 |


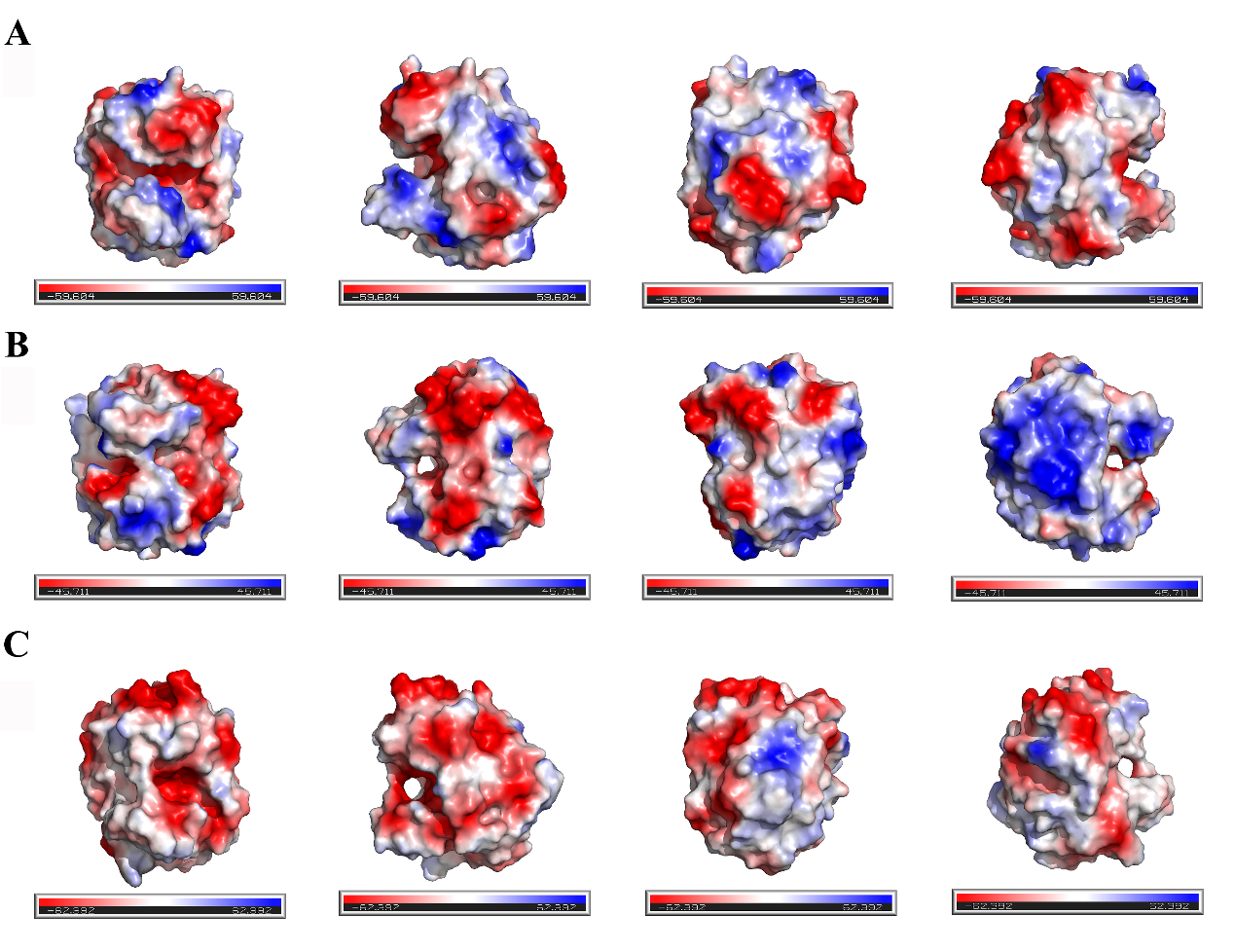


**Figure S1** Distribution of surface charge in *Tl*XynA (A), *Bs*XynJ (B) and *Ak*XynC (C). Positive charges (blue) and negative charges (red) are projected onto a solvent-accessible surface. From left to right images, looking directly into the active site pocket, are rotated 90, 180, 270 around a vertical axis compared with the left images.


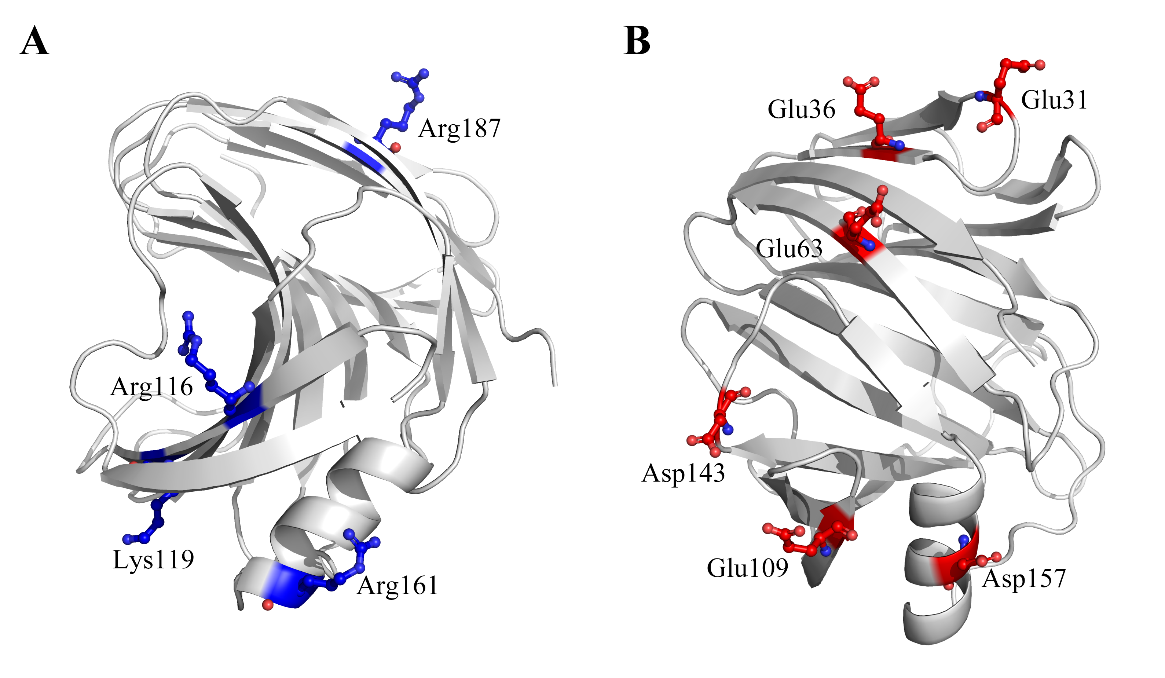


**Figure S2** Mutation sites of *Tl*XynA. Positive charges (blue, A) and negative charges (red, B) residues were displayed by stick model.

**
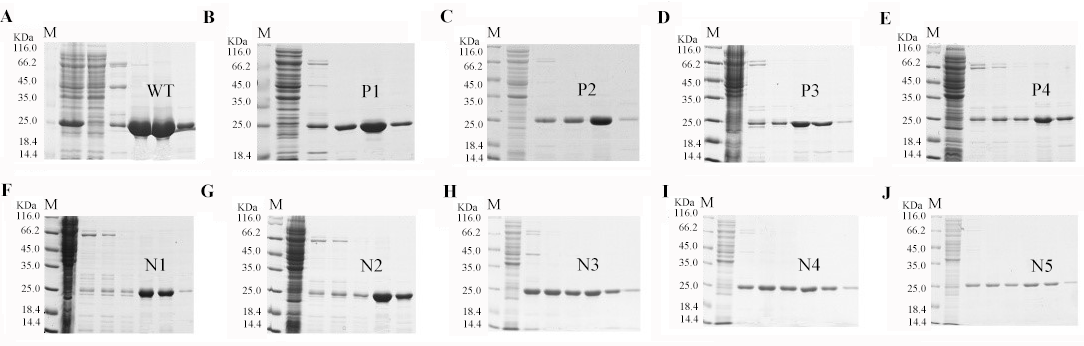
**

**Figure S3** SDS-PAGE of *Tl*XynA and its mutants. (a-j) displayed all the protein of TlXynA, P1, P2, P3, P4, N1, N2, N3, N4 and N5 respectively. All the protein heterologous expressed in *E.coli* BL21 (DE3) and purified by Ni-Sepharose columns.





**Figure S4** CD spectra of WT and mutant proteins


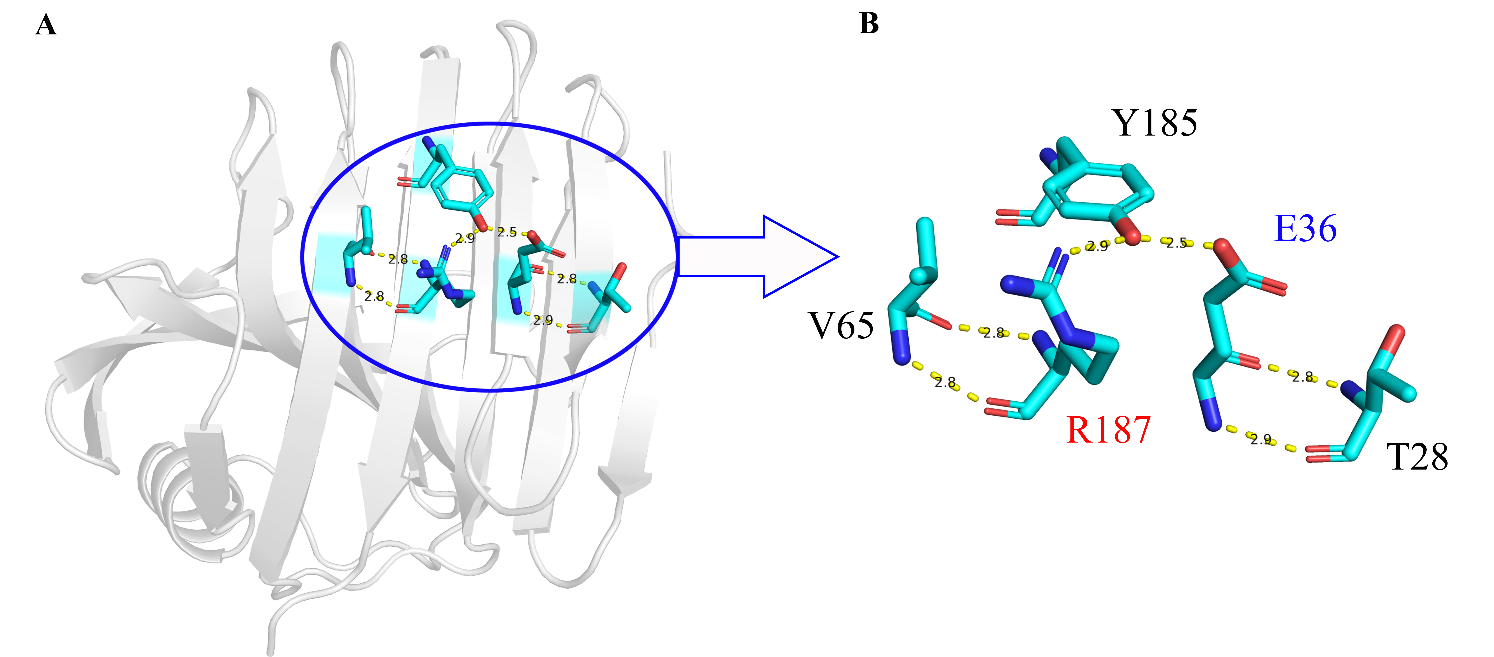


**Figure S5** Hydrogen bond network of amino acids cluster. (A) The display diagram of amino acid clusters on the overall structure (B) the amino acids position of R187 and E36 in the hydrogen bond network.
